# Hypoxia protects against the cell death triggered by oxovanadium–galactomannan complexes in HepG2 cells

**DOI:** 10.1186/s11658-019-0135-3

**Published:** 2019-03-22

**Authors:** Monique Meyenberg Cunha-de Padua, Guilhermina Rodrigues Noleto, Carmen Lucia de Oliveira Petkowicz, Silvia Maria Suter Correia Cadena, Frédéric Bost, Jacques Pouysségur, Nathalie M. Mazure

**Affiliations:** 1 Department of Biochemistry and Molecular Biology, Federal University of Parana, Curitiba, Brazil; 2 Institute for Research on Cancer and Aging of Nice, CNRS-UMR 7284-Inserm U1081, University of Nice Sophia-Antipolis, Centre Antoine Lacassagne, 33 Ave. de Valombrose, 06189 Nice, France; 3 Present Address: INSERM U1065, C3M, 151 Route de St Antoine de Ginestière, BP2 3194, 06204 Nice Cedex 03, France; 4 Medical Biology Department, Centre Scientifique de Monaco (CSM), Monaco, Monaco

**Keywords:** Hepatocellular carcinoma, Hypoxia, MSAGM:VO, Polysaccharides

## Abstract

**Background:**

Polysaccharides from various sources have been used in traditional medicine for centuries. The beneficial pharmacological effects of plant-derived polysaccharides include anti-tumor activity.

**Methods:**

Here, we evaluated the anti-cancer effect of the MSAGM:VO complex under hypoxic conditions (1% oxygen). MSAGM:VO is a complex of the hydrolysate of galactomannan (MSAGM) from *Schizolobium amazonicum* with oxovanadium (IV/V). The hepatocellular carcinoma (HCC) cell line HepG2 was selected as HCC are one of the most hypoxic solid tumors.

**Results:**

Our results showed that the strong apoptotic activity of MSAGM:VO observed in HepG2 cells under normoxic conditions was completely lost under hypoxic conditions. We found a dynamic balance between the pro- and anti-apoptotic members of the Bcl-2 protein family. The expressions of anti-apoptotic Mcl-1 and Bcl-X_L_ increased in hypoxia, whereas the expression of pro-apoptotic Bax decreased. MSAGM:VO strongly induced autophagy, which was previously characterized as a pro-survival mechanism in hypoxia. These results demonstrate total elimination of the anti-cancer activity of MSAGM:VO with activation of autophagy under conditions of hypoxia.

**Conclusion:**

Although this study is a proof-of-concept of the impact of hypoxia on the potential of polysaccharides, further study is encouraged. The anti-tumor activity of polysaccharides could be achieved in normoxia or through raising the activity of the immune system. In addition, combination strategies for therapy with anti-autophagic drugs could be proposed.

## Background

Polysaccharides (glycans) from various sources, including plants, have been extensively studied for various applications in medicine. Several possess immunomodulation properties, and can thus be classified as biological response modifiers, while others show anti-cancer effects [1, 2].

The main sources of anti-tumor polysaccharides are the cell walls of fungi [3], which are composed of structural polysaccharides such as chitin or cellulose, and a matrix of β-glucans and glycoproteins or even neutral polysaccharides such as galactomannans. Galactomannans are also present in the lichen *Ramalina celastri* [4] and the plant *Schizolobium amazonicum* [5]. The pharmacological importance of this polysaccharide type has yet to be investigated thoroughly, in particular its anti-tumor potential.

The considerable structural flexibility of polysaccharides allows a variety of chemical modifications [6]. Their unique characteristics include redox activity, variable coordination modes and reactivity towards organic substrates. In the last few decades, improving their anti-cancer potential through chemical modification has attracted increasing attention [7]. Several research groups have focused on the possibilities of complexing the substances to metals [8–11].

Vanadium, a trace metal that is essential for many species, including humans, has long been of interest in cancer therapy [12]. The first report of its anti-neoplastic effects was published in 1965 [13]. Its anti-cancer activity involves inhibition of cell proliferation, arrest of cell cycle, induction of apoptosis and DNA cleavage [14, 15]. Numerous vanadium-based compounds have been described for their capabilities to prevent and/or to reduce chemically-induced pre-neoplastic and neoplastic development in various target organs, such as breast, central nervous system, colon, liver, hematological system and connective tissue [16].

Hepatocellular carcinoma (HCC) is the fifth most common tumor worldwide and shows increasing incidence. It is a primary malignancy of the liver and it generally leads to death within 6 to 20 months [17]. HCC is unique among cancers because 90% of HCCs develop in the context of chronic liver disease and cirrhosis. Hepatocarcinogenesis is a complex multistep process. It is likely that there are several different events or series of events that can eventuate in HCC, including, inflammation, regeneration, proliferation and genetic mechanisms. Early detection is essential for a good prognosis and optimal treatments require a multidisciplinary approach combined with new therapies.

As polysaccharides may have anti-cancer activity, we investigated MSAGM:VO, which is a hydrolysate of a galactomannan preparation from *S. amazonicum* seeds complexed with oxovanadium (IV/V), for its cytotoxic effects on HepG2 cell, a human liver cancer cell line [5]. We reported inhibition of cell respiration, a decrease in membrane potential and an increase in the production of reactive oxygen species levels after 72 h of treatment. This showed perturbation of the metabolic functions of these cells in normoxia [18].

Hypoxia, a low level of oxygen, is known to inhibit cell death [19–21]. The aim of this study was to evaluate if the cytotoxic effects of MSAGM:VO were maintained in hypoxia.

## Materials and methods

### Cell culture

HepG2 is a human hepatocellular carcinoma cell line. HepG2 cells were grown in Dulbecco’s modified Eagle’s medium (DMEM, Gibco) supplemented with 10% fetal bovine serum (FBS), penicillin (10 U/ml) and streptomycin (10 μg/ml). For normoxia, the cells were maintained in a humidified incubator at 37 °C with 5% CO_2_ and in air. For hypoxia, cells were transferred to an *Invivo*_*2*_ hypoxic workstation (Baker Ruskinn Global) set at 37 °C, 5% CO_2_ and 1% O_2_.

### Galactomannan solution

Galactomannan from *S. amazonicum* seeds was isolated as previously described by Cunha-De Padua et al. [5]. A hydrolysate (MSAGM) in a complex with oxovanadium (MSAGM:VO) was obtained as described by Cunha-De Padua et al. [18]. Polymers were diluted in ultrapure water at a concentration of 5 mg/ml and then sterilized by filtration through a 0.22 μm membrane. The galactomannan solutions were kept at − 20 °C and then diluted in DMEM to perform the experiments. HepG2 cells were cultured at 50% confluence and treated the following day with MSAGM (250 μg/ml) and MSAGM:VO (250 μg/ml).

### Cell counting for viability and assessment of proliferation

Cells were plated at 100,000 cells/well and treated the following day. At determined times, cells were detached using trypsin–EDTA, suspended in their conditioned medium and evaluated for viability and proliferation, using an automatic cell counter (Advanced Detection Accurate Measurement system, Digital Bio, NanoEnTek Inc.).

### Caspase activation

Quantification of the caspase-3 and -7 activity was done using a luciferin/luciferase-based assay (Caspase-Glo 3/7 kit, Promega) according to the manufacturer’s instructions. Each condition was performed eight times and the entire experiment was done three times. Significant differences are based on Student’s *t* test *p < 0.1, **p < 0.01, ***p < 0.001.

### Immunoblotting

Cells were lysed in 1.5x SDS buffer and the protein concentration determined using the BCA assay. 40 μg of protein of whole cell extracts was resolved via SDS-PAGE and transferred onto a PVDF membrane (Millipore). Membranes were blocked in 5% non-fat milk in TN buffer (50 mM Tris-HCl pH 7.4, 150 mM NaCl) and incubated in the presence of the primary and then secondary antibodies in 5% non-fat milk in TN buffer.

The Bax and Bcl-X_L_ antibodies were purchased from Santa Cruz Biotechnology, and Mcl-1 from Sigma. The VDAC1 antibody (ab15895) was purchased from Abcam. Rabbit polyclonal anti-HIF-1α antibody (antiserum 2087) was produced and characterized in our laboratory [22]. Rabbit polyclonal anti-LC3 antibody (antiserum) was produced and characterized in our laboratory [23]. ECL signals were normalized to β-tubulin. After washing in TN buffer containing 1% Triton-X100 and then in TN buffer, immunoreactive bands were visualized with the ECL system (Amersham Biosciences).

### Immunofluorescence

For immunofluorescence, cells were grown on glass coverslips, fixed in 3.3% paraformaldehyde for 30 min at room temperature (RT), permeabilized with 0.2% Triton X-100 for 5 min, blocked with phosphate-buffered saline (PBS) containing 0.2% gelatin and 2% bovine serum albumin (BSA) for 30 min at RT, and incubated with anti-mouse cytochrome C (1/500 BD Bioscience) antibody in PBS 2% BSA for 3 h at RT. After washing, cells were incubated in the presence of a biotinylated anti-mouse secondary antibody conjugated to Alexa 594 (1:400) for 1 h at RT. After washing, coverslips were mounted in Cytifluor (Amersham Biosciences), detection of the fluorescence was performed with a Leica DMR fluorescence microscope, and images were recorded using RSImage software.

### Statistics

All values are the means ± SEM. Statistical analysis was performed using Student’s *t* test via the Microsoft Excel app. The *p* values are indicated. All categorical data used numbers and percentages. Quantitative data are presented using the median and range or mean.

## Results

We hypothesized that hypoxia protects HepG2 cells against apoptosis induced by MSAGM:VO. We investigated drug resistance in HepG2 cells in normoxia (21% O_2_) compared to hypoxia (1% O_2_). MSAGM was used as a control.

**Fig. 1:**
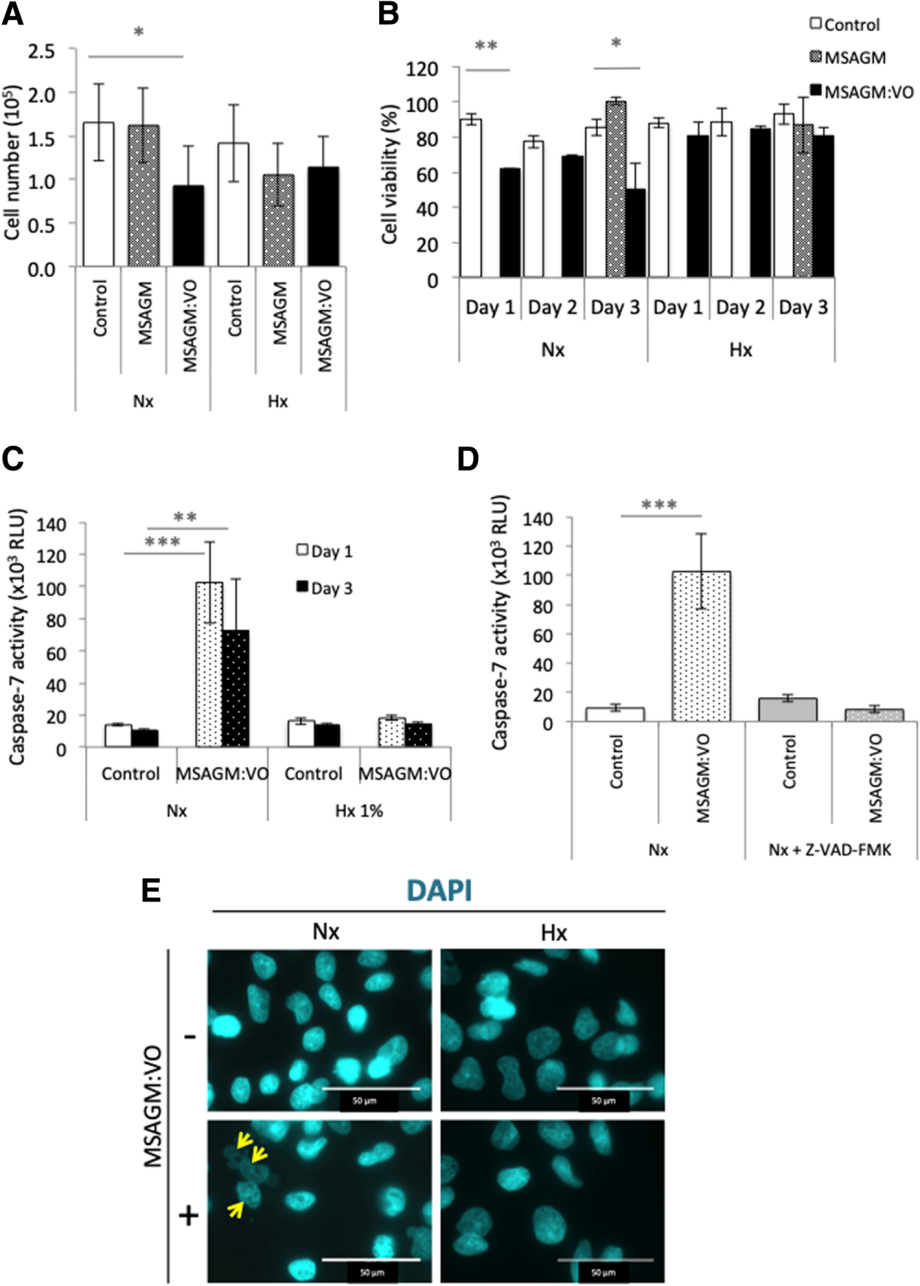
MSAGM:VO induced apoptosis in normoxia but not in hypoxia in HepG2 cells. **a** Cells were seeded at the same density and incubated in normoxia (Nx) or hypoxia (Hx 1%) in the absence or presence of MSAGM or MSAGM:VO for 72 h. The means ± SEM are representative of three independent experiments carried out in duplicate. **b** Cell viability (%) of HepG2 cultured in the absence (Control), or presence of MSAGM or MSAGM:VO for 24 h (day 1), 48 h (day 2) or 72 h (day 3) in (Nx) or (Hx) was measured with an automatic cell counter. **c** HepG2 cells were incubated in Nx or Hx 1% for 24 h (day 1) or 72 h (day 3) and challenged with MSAGM or MSAGM:VO. Apoptosis was evaluated from the level of caspase-7. **d** HepG2 cells were incubated in Nx for 72 h with MSAGM:VO in the absence or presence of Z-VAD-FMK, an inhibitor of apoptosis. Apoptosis was evaluated from the level of caspase-7. **e** HepG2 cells were incubated in Nx or Hx for 72 h and challenged with MSAGM:VO. Cells were stained with DAPI (blue) to highlight the nucleus and its morphology. Arrows show blebbing in HepG2 cells in Nx in the presence of MSAGM:VO. DAPI: 4′,6-diamidino-2-phenylindole; RLU: relative luciferase units. *p < 0.05, **p < 0.005, ***p < 0.0005

We first tested if MSAGM:VO (250 μg/ml) could modify HepG2 proliferation over three days of treatment (Fig. 1a). MSAGM:VO only statistically decreased proliferation in normoxia.

We then tested MSAGM:VO for its capacity to trigger cell death after 24 (day 1), 48 (day 2) and 72 h (day 3). The basal level of cell viability was similar in normoxia (90 ± 3% on day 1, 77.5 ± 3.5% on day 2 and 85.7 ± 4.7% on day 3) and hypoxia (88 ± 2%, 88 ± 7.8% and 93 ± 5.6%, respectively; Fig. 1b). However, MSAGM:VO triggered cell death in normoxia (cell viabilities of 62%, 69.5 ± 0.7% and 50.5 ± 14.8%, respectively) whereas HepG2 cells were less sensitive to MSAGM:VO-induced cell death in hypoxia (cell viabilities of 80.5 ± 5.5% on day 2 and 81 ± 3% on day 3). MSAGM alone did not trigger cell death in normoxia or hypoxia after 72 h (day 3).

To confirm that MSAGM:VO induced apoptosis in normoxia, the activity of caspase-7, an apoptosis-related cysteine peptidase, was measured (Fig. 1c, d). A low level of caspase-7 activity was detected in both normoxia and hypoxia. In normoxia, MSAGM:VO enhanced the activity by 7.8-fold after 24 h and this higher level was maintained after 3 days (6.9-fold induction). However, no increase in its activity was detected in hypoxia (Fig. 1c).

To characterize the nature of the cell death signal, Z-VAD-FMK, a potent inhibitor of apoptosis, was added in the absence or presence of MSAGM:VO in normoxia (Fig. 1d). As previously observed, MSAGM:VO-induced cell death was about 11.3-fold higher in the absence of Z-VAD-FMK, but apoptosis was fully inhibited in the presence of Z-VAD-FMK.

Apoptosis is characterized by cell shrinkage, maintenance of plasma integrity, chromatin condensation, nuclear fragmentation, and activation of caspases. We observed changes in the cell morphology with the nucleic acid stain 4′,6′-diamidino-2-phenylindole (DAPI). DAPI-stained live cells were visible in normoxia and hypoxia in the absence of MSAGM:VO (Fig. 1e). Chromatin condensation (yellow arrows in Fig. 1e) was visible with DAPI in normoxia in the presence of MSAGM:VO. No chromatin condensation was observed in hypoxia.

These results confirm that MSAGM:VO induced apoptosis in HepG2 cells under normoxia. However, hypoxia promoted a resistance to this MSAGM:VO-induced cell death.

**Fig. 2:**
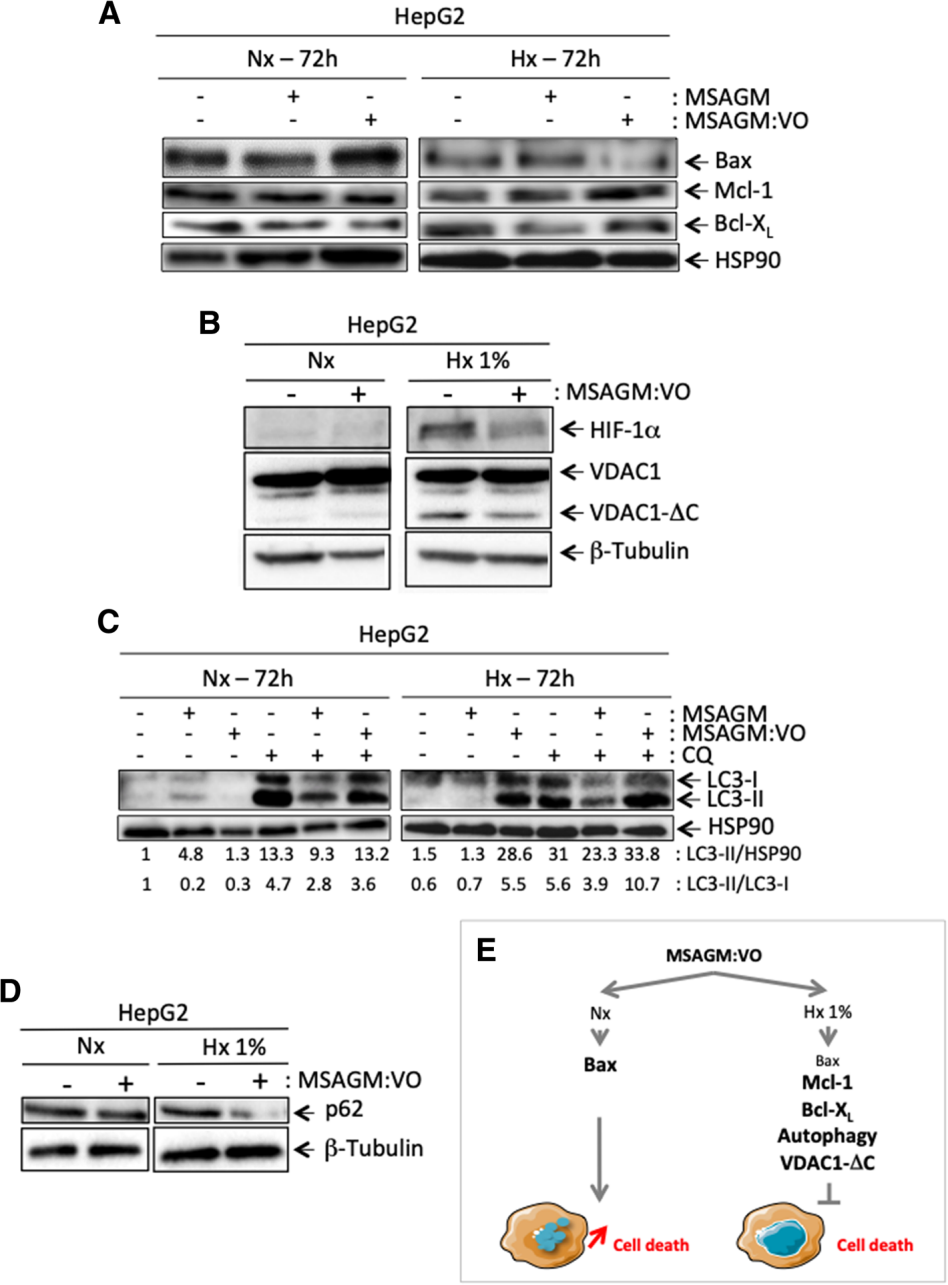
Mechanisms activated in hypoxia protect cells from the apoptosis triggered by MSAGM:VO. **a** and **b** HepG2 were incubated in Nx or Hx 1% for 72 h in the absence (−) or presence (+) of MSAGM or MSAGM:VO. Cell lysates were analyzed by immunoblotting for (**a**) Bax, Mcl-1 and Bcl-X_L_ and (**b**) HIF-1α and VDAC1. HSP90 and β-tubulin were used as a loading control. **c** HepG2 cells were incubated in Nx or Hx (1% O_2_) for 72 h in the absence (−) or presence (+) of MSAGM or MSAGM:VO and in the absence (−) or presence (+) of chloroquine (CQ). Cell lysates were analyzed via immunoblotting for LC3-I and -II. HSP90 was used as a loading control. **d** HepG2 cells were incubated in Nx or Hx 1% for 72 h in the absence (−) or presence (+) of MSAGM:VO. Cell lysates were analyzed via immunoblotting for p62. β-tubulin was used as a loading control. **e** Schematic representation of a pathway for hypoxic protection of HepG2 cells in the presence of MSAGM:VO

To better understand the molecular mechanisms behind this resistance, we compared the normoxic and hypoxic levels of anti- and pro-apoptotic proteins of the Bcl-2 family in the absence or presence of MSAGM:VO (Fig. 2a). The expression of Bax, a pro-apoptotic Bcl-2-family protein, strongly increased in normoxia in the presence of MSAGM:VO, but it decreased in hypoxia. The expressions of Mcl-1 and Bcl-X_L_, two anti-apoptotic Bcl-2-family members, were strongly enhanced in hypoxia. MSAGM, used as a control, did not affect the expressions of Bax, Mcl-1 or Bcl-X_L_.

Since we previously found that the production of the truncated form of VDAC1 (VDAC1-ΔC) was associated with increased resistance to staurosporine-induced apoptosis [21, 24], we also examined the levels of VDAC1 and VDAC1-ΔC in normoxia and hypoxia in the presence of MSAGM:VO (Fig. 2b). We detected VDAC1-ΔC in cells in hypoxia with or without MSAGM:VO treatment. In hypoxia, HepG2 cells presented enlarged mitochondria with cytochrome C sequestrated within them (data not shown). The former is a prerequisite to the production of VDAC1-ΔC and the latter to resistance to apoptosis.

Finally, as hypoxia-induced autophagy triggers survival [23], we checked the potential role of autophagy in protecting HepG2 cells against MSAGM:VO-induced apoptosis by looking at LC3 (microtubule-associated protein 1 light chain 3). This protein is recruited from the cytosol in the form LC3-I to the autophagosomal membrane where it is lipidated to LC3-II (Fig. 2c). We also assessed p62, which is a protein adaptator that links LC3 and ubiquitinated substrates (Fig. 2d). Although hypoxia either did not induce or only slightly induced autophagy, MSAGM:VO treatment in hypoxia lead to an increase in the amount of LC3-II (Fig. 2c). As expected, chloroquine (CQ) treatment lead to an increase in the amount of LC3-II. This increase was substantiated in hypoxia and in the presence of MSAGM:VO, which argues strongly in favor of hypoxia- and MSAGM:VO-induced autophagic flux. Moreover, MSAGM:VO treatment under hypoxic conditions decreased strongly p62 expression (Fig. 2d).

These results demonstrate that hypoxia (1% O_2_) protects HepG2 cells from cell death by: (i) creating a balance between pro- and anti-apoptotic proteins; (ii) causing the mitochondria to enlarge; (iii) producing VDAC1-ΔC; and (iv) triggering autophagy (Fig. 2e).

## Discussion

According to an old Japanese story, the wild monkeys on Mount Fuji rarely develop cancer, high blood pressure or diabetes [25]. The story suggests that this could be to some extent related to their consumption of wild mushrooms. However, it does not reveal whether the monkeys live at the bottom of the mountain in a 21% O_2_ atmosphere or at the top with just 13% O_2_. The protection against cancer offered by the mushrooms would undoubtedly be different depending on this!

The reprogramming of metabolic pathways in a hypoxic microenvironment allows cancer cells to survive, adapt, or even escape from the constraints of their environment [26]. Apoptosis, an efficient programmed cell death that requires coordinated activation of signaling pathways and executioner proteases, is suppressed in hypoxia. However, this affirmation remains controversial and probably depends on the duration and the severity of the hypoxia.

Hypoxia regulates a variety of Bcl-2 family members with both pro- and anti-apoptotic consequences. Induction of the pro-apoptotic proteins Noxa [27], BNIP3 and/or BNIP3L [28, 29] have been reported. However, as we clearly established previously, both BNIP3 and BNIP3L are required for optimal induction of autophagy in hypoxia in a survival context [23]. Most reports are in favor of hypoxia in protecting cancer cells from apoptotic cell death. Hypoxia increases the levels of anti-apoptotic member proteins, such as Bcl-2, Bcl-X_L_ and Mcl-1, whereas it decreases the levels of pro-apoptotic Bax, Bak, Bid and Bad [30–35]. Our results also align with this way of thinking, as we did not observe apoptosis in hypoxic conditions, but did observe a decrease in Bax levels. MSAGM:VO increased the Mcl-1 and Bcl-X_L_ levels, which partially explains the survival of HepG2 cells in hypoxia. It remains to be determined how MSAGM:VO can induce anti-apoptotic proteins in hypoxia but induce apoptosis through upregulation of Bax and caspase-7 in normoxia. It is clear that under the evaluated conditions, HepG2 cancer cells are protected from MSAGM:VO-induced apoptosis.

Although mitochondrial activity is diminished in hypoxia, this organelle plays a crucial role in resistance to apoptosis [36]. When going from normoxia to hypoxia, the morphology of the mitochondria switches from a tubular to an enlarged network, which traps cytochrome C within the organelle [20]. VDAC1, a protein localized on the outer mitochondrial membrane, is then cleaved by an endopeptidase, providing more efficient metabolism and conferring resistance to cell death [21, 24]. We observed the production of the cleaved form of VDAC1 in HepG2 cells cultured in hypoxia in the absence or presence of MSAGM:VO, which strongly suggests that this process is also involved in the protection of HepG2 cells in hypoxia and that MSAGM:VO does not influence the mechanism through which VDAC1-ΔC is cleaved.

In 2007, Tracy et al. showed that hypoxia triggered non-apoptotic cell death in mouse embryonic fibroblasts through BNIP3 induction [37]. They demonstrated that this type of cell death involved autophagy, a catabolic process necessary for the constitutive turnover of long-lived and damaged macromolecules. Hypoxia-induced autophagy and/or cell death through BNIP3 was also described for glioma and breast cancer cells [38]. In 2009, we demonstrated that hypoxia-induced autophagy occurs in numerous normal and cancer cells in hypoxia (1% oxygen) and in severe hypoxia (0.1% oxygen) [23]. However, in this study, autophagy was not or was only slightly induced by hypoxia in HepG2 cells. However, MSAGM:VO triggered autophagy in both normoxia and hypoxia, with stronger autophagic induction in hypoxia compared to normoxia. Autophagy is generally upregulated in response to diverse stimuli, including low nutritional status, oxidative stress, microbial insult, and as previously described, low oxygen status. Few studies have shown the induction of autophagy by polysaccharides. Zhao et al. described that an extract of the mushroom *Grifola frondosa* in combination with vitamin C induced both apoptosis and autophagy in HepG2 cells, similar to the observations for MSAGM:VO [39]. However, they did not explain the molecular mechanisms involved in the process of induction of autophagy. Meng et al. also described enhanced autophagy in the presence of polysaccharides from the plant *Astragalus,* which also induced an inflammatory response [40]. They proposed that activation of the PI3K/Akt/mTOR pathway decreased when cells were treated with *Astragalus* polysaccharides, which also repressed cell growth. However, we did not detect a decrease in proliferation when HepG2 cells were treated with MSAGM:VO in hypoxia, suggesting that the mTOR pathway was not involved.

It is crucial to look for new therapies to treat HCC as it has a poor prognosis, limited therapeutic options and a high morbidity. While both the normal liver and HCC are highly vascularized, rapid growth of tumor cells within nodules scavenges a substantial amount of oxygen, often producing a hypoxic microenvironment. In fact, HCC are one of the most hypoxic tumors with the oxygen level close to 0.8% [41].

MSAGM:VO initially appeared to be a good candidate as a potential therapeutic for the treatment of HCC, but the pharmacological effect may be absent in a hypoxic microenvironment. Combination strategies for therapy with anti-autophagic drugs such as 3-methyladenine, bafilomycin or chloroquine could be proposed. Further research is required to improve the anti-tumor activity of those polysaccharides when cells are exposed to hypoxia.

## Abbreviations

BSA: Bovine serum albumin
CQ: Chloroquine
DAPI: 4′,6′-diamidino-2-phenylindole
FBS: Fetal bovine serum
HCC: Hepatocellular carcinoma
MSAGM:VO: Native galactomannan from *S. amazonicum* (SAGM) and its modified form (*MSAGM*) complexed with oxovanadium
PBS: Phosphate-buffered saline
RT: Room temperature

## References

[1] Leung MY, Liu C, Koon JC, Fung KP (2006). Polysaccharide biological response modifiers. Immunol Lett.

[2] Zong A, Cao H, Wang F (2012). Anticancer polysaccharides from natural resources: a review of recent research. Carbohydr Polym.

[3] Wasser SP (2014). Medicinal mushroom science: current perspectives, advances, evidences, and challenges. Biom J.

[4] Noleto GR, Merce AL, Iacomini M, Gorin PA, Soccol VT, Oliveira MB (2002). Effects of a lichen galactomannan and its vanadyl (IV) complex on peritoneal macrophages and leishmanicidal activity. Mol Cell Biochem.

[5] Cunha de Padua MM, Suter Correia Cadena SM, de Oliveira Petkowicz CL, Martinez GR, Rodrigues Noleto G (2017). Galactomannan from Schizolobium amazonicum seed and its sulfated derivatives impair metabolism in HepG2 cells. Int J Biol Macromol.

[6] Meng X, Liang H, Luo L (2016). Antitumor polysaccharides from mushrooms: a review on the structural characteristics, antitumor mechanisms and immunomodulating activities. Carbohydr Res.

[7] Li S, Xiong Q, Lai X, Li X, Wan M, Zhang J (2015). Molecular modification of polysaccharides and resulting bioactivities. Compr Rev Food Sci Food Saf.

[8] do Amaral AE, Petkowicz CL, Merce AL, Iacomini M, Martinez GR, Merlin Rocha ME (2015). Leishmanicidal activity of polysaccharides and their oxovanadium(IV/V) complexes. Eur J Med Chem.

[9] Kremer LE, McLeod AI, Aitken JB, Levina A, Lay PA (2015). Vanadium(V) and -(IV) complexes of anionic polysaccharides: controlled release pharmaceutical formulations and models of vanadium biotransformation products. J Inorg Biochem.

[10] Noleto GR, Petkowicz C, Merce AL, Noseda MD, Mendez-Sanchez SC, Reicher F (2009). Two galactomannan preparations from seeds from Mimosa scabrella (bracatinga): complexation with oxovanadium(IV/V) and cytotoxicity on HeLa cells. J Inorg Biochem.

[11] Winter E, Borguezani DR, ALR M, ALR M, MAL R (2014). Novel trends in Cyclodextrins: a review. Molecular and supramolecular bioinorganic chemistry. Applications in medical and environmental sciences.

[12] Leon IE, Butenko N, Di Virgilio AL, Muglia C, Baran EJ, Cavaco I (2014). Vanadium and cancer treatment: antitumoral mechanisms of three oxidovanadium(IV) complexes on a human osteosarcoma cell line. J Inorg Biochem.

[13] Kieler J, Gromek A, Nissen NI (1965). Studies on the antineoplastic effect of vanadium salts. Acta Chir Scand Suppl.

[14] Evangelou AM (2002). Vanadium in cancer treatment. Crit Rev Oncol Hematol.

[15] Ray RS, Rana B, Swami B, Venu V, Chatterjee M (2006). Vanadium mediated apoptosis and cell cycle arrest in MCF7 cell line. Chem Biol Interact.

[16] Bishayee A, Waghray A, Patel MA, Chatterjee M (2010). Vanadium in the detection, prevention and treatment of cancer: the in vivo evidence. Cancer Lett.

[17] Balogh J, Victor D, Asham EH, Burroughs SG, Boktour M, Saharia A (2016). Hepatocellular carcinoma: a review. J Hepatocell Carcinoma.

[18] Cunha-de Padua MM, Suter Correia Cadena SM, de Oliveira Petkowicz CL, Martinez GR, Merlin Rocha ME, ALR M (2017). Toxicity of native and oxovanadium (IV/V) galactomannan complexes on HepG2 cells is related to impairment of mitochondrial functions. Carbohydr Polym.

[19] Pouyssegur J, Dayan F, Mazure NM (2006). Hypoxia signalling in cancer and approaches to enforce tumour regression. Nature.

[20] Chiche J, Rouleau M, Gounon P, Brahimi-Horn MC, Pouyssegur J, Mazure NM (2010). Hypoxic enlarged mitochondria protect cancer cells from apoptotic stimuli. J Cell Physiol.

[21] Brahimi-Horn MC, Ben-Hail D, Ilie M, Gounon P, Rouleau M, Hofman V (2012). Expression of a truncated active form of VDAC1 in lung cancer associates with hypoxic cell survival and correlates with progression to chemotherapy resistance. Cancer Res.

[22] Richard DE, Berra E, Gothie E, Roux D, Pouyssegur J (1999). p42/p44 mitogen-activated protein kinases phosphorylate hypoxia- inducible factor 1alpha (HIF-1alpha) and enhance the transcriptional activity of HIF-1. J Biol Chem.

[23] Bellot G, Garcia-Medina R, Gounon P, Chiche J, Roux D, Pouyssegur J (2009). Hypoxia-induced autophagy is mediated through hypoxia-inducible factor induction of BNIP3 and BNIP3L via their BH3 domains. Mol Cell Biol.

[24] Brahimi-Horn MC, Lacas-Gervais S, Adaixo R, Ilc K, Rouleau M, Notte A (2015). Local mitochondrial-endolysosomal microfusion cleaves the voltage-dependent anion channel 1 to promote survival in hypoxia. Mol Cell Biol.

[25] Daba AS, Ezeronye OU (2003). Anti-cancer effect of polysaccharides isolated from higher basidiomycetes mushrooms. Afric J Biotech.

[26] Eales KL, Hollinshead KE, Tennant DA (2016). Hypoxia and metabolic adaptation of cancer cells. Oncogene.

[27] Kim JY, Ahn HJ, Ryu JH, Suk K, Park JH (2004). BH3-only protein Noxa is a mediator of hypoxic cell death induced by hypoxia-inducible factor 1alpha. J Exp Med.

[28] Bruick RK (2000). Expression of the gene encoding the proapoptotic Nip3 protein is induced by hypoxia. Proc Natl Acad Sci U S A.

[29] Sowter HM, Ratcliffe PJ, Watson P, Greenberg AH, Harris AL (2001). HIF-1-dependent regulation of hypoxic induction of the cell death factors BNIP3 and NIX in human tumors. Cancer Res.

[30] Chen N, Chen X, Huang R, Zeng H, Gong J, Meng W (2009). BCL-xL is a target gene regulated by hypoxia-inducible factor-1{alpha}. J Biol Chem.

[31] Erler JT, Cawthorne CJ, Williams KJ, Koritzinsky M, Wouters BG, Wilson C (2004). Hypoxia-mediated down-regulation of bid and Bax in tumors occurs via hypoxia-inducible factor 1-dependent and -independent mechanisms and contributes to drug resistance. Mol Cell Biol.

[32] Liu XH, Yu EZ, Li YY, Kagan E (2006). HIF-1alpha has an anti-apoptotic effect in human airway epithelium that is mediated via Mcl-1 gene expression. J Cell Biochem.

[33] Palladino MA, Shah A, Tyson R, Horvath J, Dugan C, Karpodinis M (2012). Myeloid cell leukemia-1 (Mc1-1) is a candidate target gene of hypoxia-inducible factor-1 (HIF-1) in the testis. Reprod Biol Endocrinol.

[34] Sasabe E, Tatemoto Y, Li D, Yamamoto T, Osaki T (2005). Mechanism of HIF-1alpha-dependent suppression of hypoxia-induced apoptosis in squamous cell carcinoma cells. Cancer Sci.

[35] Sharma M, Machuy N, Bohme L, Karunakaran K, Maurer AP, Meyer TF (2011). HIF-1alpha is involved in mediating apoptosis resistance to chlamydia trachomatis-infected cells. Cell Microbiol.

[36] Mazure NM, Brahimi-Horn MC, Pouyssegur J (2011). Hypoxic mitochondria: accomplices in resistance. Bull Cancer.

[37] Tracy K, Dibling BC, Spike BT, Knabb JR, Schumacker P, Macleod KF (2007). BNIP3 is an RB/E2F target gene required for hypoxia-induced autophagy. Mol Cell Biol.

[38] Azad MB, Chen Y, Henson ES, Cizeau J, McMillan-Ward E, Israels SJ (2008). Hypoxia induces autophagic cell death in apoptosis-competent cells through a mechanism involving BNIP3. Autophagy.

[39] Zhao F, Zhao J, Song L, Zhang YQ, Guo Z, Yang KH (2017). The induction of apoptosis and autophagy in human hepatoma SMMC-7721 cells by combined treatment with vitamin C and polysaccharides extracted from Grifola frondosa. Apoptosis.

[40] Meng Q, Meng Q, Du X, Wang H, Gu H, Zhan J, Zhou Z (2017). Astragalus polysaccharides inhibits cell growth and pro-inflammatory response in IL-1beta-stimulated fibroblast-like synoviocytes by enhancement of autophagy via PI3K/AKT/mTOR inhibition. Apoptosis.

[41] McKeown SR (2014). Defining normoxia, physoxia and hypoxia in tumours-implications for treatment response. Br J Radiol.

